# Elevated alanine transaminase in liver transplant recipients after BNT162b2 vaccination: a cohort study

**DOI:** 10.1038/s41541-025-01233-7

**Published:** 2025-08-02

**Authors:** Jacob Siewertsen Bergmann, Sebastian Rask Hamm, Louise Bering, Christian Ross Pedersen, Ask Bock, Safura-Luise Heidari, Gerda Elisabeth Villadsen, Annette Dam Fialla, Gro Linno Willemoe, Peter Holland-Fischer, Susanne Dam Nielsen

**Affiliations:** 1 Viro-Immunology Research Unit, Department of Infectious Diseases, Copenhagen University Hospital - Rigshospitalet, Copenhagen, Denmark; 2 Department of Surgical Gastroenterology and Transplantation, Copenhagen University Hospital - Rigshospitalet, Copenhagen, Denmark; 3 Department of Hepatology and Gastroenterology, Aarhus University Hospital, Aarhus, Denmark; 4 Department of Clinical Medicine, Aarhus University, Aarhus, Denmark; 5 Department of Gastroenterology and Hepatology, Odense University Hospital, Odense, Denmark; 6 Department of Pathology, Rigshospitalet, Copenhagen University Hospital, Copenhagen, Denmark; 7 Department of Gastroenterology, Aalborg University Hospital, Aalborg, Denmark; 8 Department of Clinical Medicine, Faculty of Health and Medical Sciences, University of Copenhagen, Copenhagen, Denmark

**Keywords:** RNA vaccines, Drug safety, Liver diseases, SARS-CoV-2

## Abstract

Liver transplant (LTx) recipients risk severe COVID-19. Vaccination reduces this risk. However, there may be side effects, including elevated alanine transaminase (ALT) which could lead to increased use of liver biopsy. We aimed to describe prevalence and relative incidence of elevated ALT 90 days before and after BNT162b2 vaccination in LTx recipients. Furthermore, we aimed to describe changes in prevalence of liver biopsies before and after BNT162b2 vaccination. We included 393 LTx recipients from The Danish Comorbidity in Liver Transplant Recipients (DACOLT) study. We calculated prevalence of elevated ALT and liver biopsies before and after each BNT162b2 vaccine dose. We used self-control case series (SCCS) analysis to investigate whether vaccination was associated with higher relative incidence of elevated ALT. Prevalence of elevated ALT, around each vaccine dose, was comparable. We did not find higher relative incidence of elevated ALT after vaccination. The prevalence of liver biopsies around vaccination was comparable.

## Introduction

Liver transplant (LTx) recipients have a higher risk of severe COVID-19 than the background population, even during the Omicron era^1,2^. Although LTx recipients elicit lower antibody responses to COVID-19 mRNA vaccines than immunocompetent controls, vaccination effectively lowers the risk of severe COVID-19 in this population^3–5^. In general, COVID-19 mRNA vaccines are safe in LTx recipients with reported adverse events comparable to the background population^6–8^. Hence, at present, booster vaccines against COVID-19 are recommended in LTx recipients^9–11^. However, previous case reports have reported acute graft rejection, liver damage and alanine transaminase (ALT) elevation in LTx recipients following the BNT162b2 vaccine^12–18^. Acute graft rejection is a serious complication in LTx recipients and is often suspected in LTx recipients with unexplained ALT elevation. Consequently, elevated ALT following vaccination may rise suspicion of acute graft rejection and potentially lead to further diagnostics, including a liver biopsy, and thus exposing the LTx recipients to potential complications associated with this procedure^19^. One study examined the safety in kidney and liver transplant recipients that received a two-dose series of either the BNT162b2 vaccine or the ChAdOx1 vaccine^20^. This study observed no changes in mean ALT among LTx recipients before the first vaccination compared to 2 weeks after second vaccination. However, there are no studies investigating changes in ALT beyond the first two vaccine doses and there are no previous reports on associations between BNT162b2 vaccine and use of liver biopsies. We hypothesized that BNT162b2 vaccination is associated with increased risk of ALT elevations and increased use of liver biopsies in LTx recipients. In this study, we aimed to investigate whether BNT162b2 vaccination is associated with ALT elevation and increased use of liver biopsies in LTx recipients. Furthermore, we aimed to explore whether the relative incidence of ALT elevation is higher after BNT162b2 vaccinations in LTx recipients.

## Results

We included 393 participants in this study. At baseline, the median age was 54.3 years (interquartile range (IQR) 43.6–63.8) and 46.1% were female. The median time since transplantation was 7.3 years (IQR 4.0–13.7). At baseline, 244 (62.1%) had an ALT measurement, 30 (12.3%) of those had elevated ALT. Clinical characteristics are shown in Table 1.

**Table 1:** Baseline characteristics

Participants, *n*	393
Age in years, median (IQR)	54.3 (43.6–63.8)
Female sex, %	46.1
Years since transplantation, median (IQR)	7.3 (4.0–13.7)
Reason for transplantation^a^	
- Autoimmune liver disease^b^, %	46.3
- Alcoholic or cryptogenic cirrhosis, %	17.8
- Hepatocellular carcinoma, %	6.9
- Fulminant hepatic failure, %	7.4
- Metabolic disease, %	5.9
- Hepatitis C, %	2.8
- Other, %	22.4
Immunosuppressive medication at inclusion	
Current use of antimetabolites	
- Mycophenolate, %	66.9
- Azathioprine %	13.5
- No antimetabolites, %	19.6
- Corticosteroids %	40.7
Current use of calcineurin inhibitor	
- Tacrolimus, %	80.4
- Ciclosporin %	11.2
Current use of mTOR inhibitor (Everolimus), %	7.6
Acute graft rejection within six months before study, n	1
Participants with at least one acute graft rejection during follow-up, n	3
Number of BNT162b2 vaccines, *n* (%)	
- 0	15 (3.8)
- 1	1 (0.3)
- 2	17 (4.3)
- 3	50 (12.7)
- 4	53 (13.5)
- 5	87 (22.1)
- 6	169 (43.0)
- 7	1 (0.3)
Elevated ALT at baseline, *n* (%)	30 (12.3)
Missing ALT measurements at baseline, *n* (%)	149 (37.9)
End of follow-up during study period, *n* (%)	
- Died	24 (6.1)
- Different vaccine than BNT162b2	10 (2.5)
▪ mRNA-1237	6 (1.5)
▪ChAdOX1	4 (1.0)
- Re-transplanted	4 (1.0)

*ALT* Alanine transaminase, *IQR* Interquartile range, *mTOR* The mammalian target of rapamycin

^a^The liver transplant recipient may have more than one reason for undergoing transplantation

^b^Autoimmune liver disease comprises: Primary sclerosing cholangitis (30.8%), autoimmune hepatitis (10.2%), primary biliary cholangitis (9.2%), and other autoimmune liver disease (0.5%).

At the end of follow-up one participant (0.3%) had received seven doses of a BNT162b2 vaccine, 169 (43.0%) had received six doses, 87 (22.1%) had received five doses, 53 (13.5%) had received four doses, 50 (12.7%) had received three doses, 17 (4.3%) had received two doses, 1 (0.3%) had received one dose and 15 (3.8%) had not received any BNT162b2 vaccines. The reasons for end of follow-up before the end of the study period was, re-transplantation (*n* = 4, 1%), death (*n* = 24, 6.1%), and administration of a different COVID-19 vaccine than a monovalent or bivalent BNT162b2 vaccine (*n* = 10, 2.5%) (Table 1). Among participants who received other vaccines than BNT162b2, four received ChAdOX1 and six received mRNA-1237. In a sensitivity analysis, we excluded the 10 participants who were censored due to receiving other COVID-19 vaccines than monovalent or bivalent BNT162b2 and found all results to be robust. No deaths during follow-up were related to COVID-19 or COVID-19 vaccination.

### The prevalence of elevated ALT

There was no significant difference in prevalence of elevated ALT before the first and after second vaccine dose (Table 2). Likewise, there was no difference in prevalence of elevated ALT before and after the third vaccine dose, before and after the fourth vaccine dose, before and after the fifth vaccine dose, or before and after the sixth vaccine dose (Table 2).

**Table 2:** Differences in prevalence of elevated ALT before and after each vaccine dose

Time period	Sample count, *n*	Missing samples at each dose, *n* (%)^a^	Prevalence of elevated ALT, *n* (%)	95% CI	Difference in prevalence, pp (95% CI)
Before first vaccine	298	79 (21.0)	16 (5.4)	3.1–8.6	+2.3 % (−1.1–5.9, *p* = 0.230)
After second vaccine			23 (7.7)	5.0–11.6	
Before third vaccine	207	153 (42.5)	15 (7.2)	4.1–11.7	+0.5 % (−3.8–4.8, *p* = 1)
After third vaccine			16 (7.7)	4.5–12.2	
Before fourth vaccine	71	239 (77.1)	8 (11.3)	5.0–21.0	+2.8 % (−5.1–11.1, *p* = 0.683)
After fourth vaccine			10 (14.1)	7.0–24.4	
Before fifth vaccine	182	75 (29.2)	13 (7.1)	3.9–11.9	−2.7% (−7.5–1.7, *p* = 0.302)
After fifth vaccine			8 (4.4)	1.9–8.5	
Before sixth vaccine	135	35 (20.6)	8 (5.9)	2.6–11.3	+0.7 (−3.9–5.5, *p* = 1)
After sixth vaccine			9 (6.7)	3.1–12.3	

*ALT* Alanine transaminase, *pp* percentage points.

^a^Missing samples at each dose represents participants who received the respective vaccine but were excluded from analyses if they lacked an ALT sample both within 90 days pre- and post-vaccination, or if vaccination occurred less than 180 days after a previous dose.

**Fig. 1:**
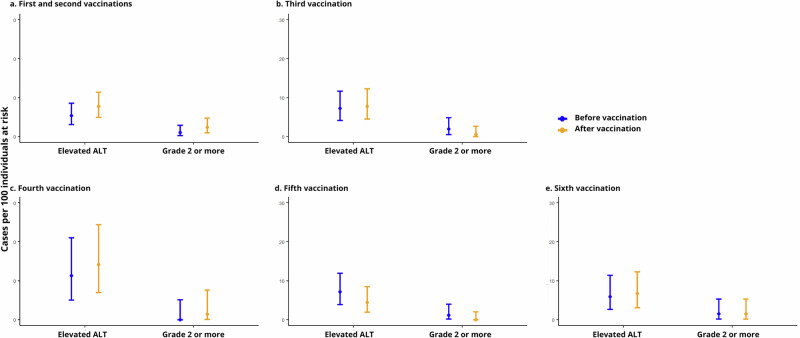
Prevalence of elevated ALT 90 days before and after each vaccine dose. **a** Prevalence of cases of elevated ALT and cases of grade ≥2 elevated ALT 90 days before the first vaccine dose (blue circle) and 90 days after the second vaccine dose (yellow circle). The whiskers indicate 95% confidence intervals. **b**–**e** Corresponding data before and after the third to sixth vaccine doses, respectively.

Figure 1 presents the prevalence of elevated ALT, regardless of common terminology criteria for adverse events (CTCAE) grade, before and after each vaccine dose. Prevalence of elevated ALT above grade 1 before and after each vaccine dose are shown in Table 3. Supplementary Table 1 summarizes the distribution of all ALT elevations by grade 90 days before and after each vaccine dose.

**Table 3:** Difference in prevalence of elevated ALT above grade 1 before and after each vaccine dose

Time period	Sample count, *n*	Missing samples at each dose, *n* (%)^a^	Prevalence of elevated ALT above grade 1, *n* (%)	95% CI	Difference in prevalence, pp (95% CI)
Before first vaccine	298	79 (21.0)	3 (1.0)	0.2–2.9	+1.3% (−0.8–3.8, *p* = 0.289)
After second vaccine			7 (2.3)	0.9–4.8	
Before third vaccine	207	153 (42.5)	4 (1.9)	0.5–4.9	−1.4% (−4.3–0.8, *p* = 0.248)
After third vaccine			1 (0.5)	0.01–2.7	
Before fourth vaccine	71	239 (77.1)	0 (0.0)	0.0–5.1	+1.4% (−3.9–7.6, *p* = 1)
After fourth vaccine			1 (1.4)	0.04–7.6	
Before fifth vaccine	182	75 (29.2)	2 (1.1)	0.1–3.9	−1.1% (−3.9–1.1, *p* = 0.480)
After fifth vaccine			0 (0.0)	0.0–2.0	
Before sixth vaccine	135	35 (20.6)	2 (1.5)	0.2–5.3	0.0% (−3.7–3.7, *p* = 1)
After sixth vaccine			2 (1.5)	0.2–5.3	

*ALT* Alanine transaminase, *pp* percentage points.

^a^Missing samples at each dose represents participants who received the respective vaccine but were excluded from analyses if they lacked an ALT sample both within 90 days pre- and post-vaccination, or if vaccination occurred less than 180 days after a previous dose.

### The incidence of positive SARS-CoV-2 polymerase chain reaction test

To investigate the potential for confounding due to SARS-CoV-2 infection status, we calculated the incidence rate of positive SARS-CoV-2 polymerase chain reaction (PCR) test in the 90 days before and after vaccination with BNT162b2. Before vaccination, 46 cases of positive SARS-CoV-2 PCR tests were detected during 334 person-years of follow-up, resulting in an incidence rate of 0.14 (95% confidence interval (CI): 0.10–0.19) per person-year. After vaccination, 86 cases were detected during 371 person-years of follow-up, resulting in an incidence rate of 0.24 (95% CI: 0.19–0.29) per person-year.

### Relative incidence of elevated ALT

During the study period, 393 LTx recipients contributed with 1325 person-years of follow-up (PYFU). We observed a total of 514 elevated ALT measurements corresponding to an incidence rate of 0.39 per PYFU (95% CI: 0.36–0.42). The 514 elevated ALT measurements were observed in 180 participants who were included in the self-controlled case series (SCCS) analysis and contributed with 435 PYFU in control periods and 183 PYFU in risk periods. The incidence rate ratio (IRR) for elevated ALT in risk periods compared to control periods was 1.1 (95% CI: 0.9–1.3, *p* = 0.306).

Of the 514 elevated ALT measurements, 444 (86.4%) were grade 1, 37 (7.2%) were grade 2, 29 (5.6%) were grade 3 and four (0.8%) were grade 4. Among the 180 individuals with elevated ALT measurements, the highest grade observed throughout the study period was grade 1 in 132 (73.3%) individuals, grade 2 in 23 (12.8%) individuals, grade 3 in 21 (11.7%) individuals and grade 4 in four (2.2%) individuals. We found that events with ALT elevations ≥ grade 2 lead to changes in immunosuppressive maintenance therapy in 12 cases, and to hospitalization in five cases.

### The prevalence of biopsies

**Fig. 2:**
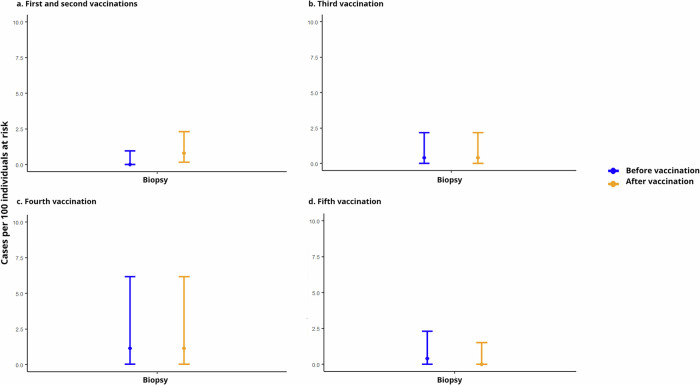
Prevalence of biopsies 90 days before and after each vaccine dose. **a** Prevalence of biopsies performed on clinical indication 90 days before the first vaccine dose (blue circle) and 90 days after the second vaccine dose (yellow circle). The whiskers indicate 95% confidence intervals. **b–d** Corresponding data before and after the third to fifth vaccine doses, respectively.

When investigating the prevalence of biopsies performed on clinical indication before the first and after the second vaccine dose, we found no difference (Table 4). Furthermore, there was no difference in prevalence of biopsies performed on clinical indication before and after the third vaccine dose, before and after the fourth vaccine dose, or before and after the fifth vaccine dose (Table 4). There were no biopsies performed on clinical indication before or after the sixth vaccine dose. The prevalence of biopsies before and after each vaccine dose is shown in Fig. 2. In the five cases where liver biopsies were performed on clinical indication after vaccination, elevated ALT measurements were a part of, but not the only clinical finding leading to the biopsy. Vaccination related liver injury was not suspected by clinicians in any of the cases.

**Table 4:** Difference in prevalence of biopsies within 90 days before and after each vaccine dose

Time period	Sample count, *n*	Missing at each dose, *n* (%)^a^	Prevalence of biopsies, *n* (%)	95% CI	Difference in prevalence, *pp* (95% CI)
Before first vaccine	377	0 (0.0)	0 (0.0)	0.0–1.0	+0.8% (−0.3–2.3, *p* = 1)
After second vaccine			3 (0.8)	0.01–2.2	
Before third vaccine	252	108 (30.0)	1 (0.4)	0.01–2.2	+0 % (−1.8–1.8, *p* = 1)
After third vaccine			1 (0.4)	0.2–3.4	
Before fourth vaccine	88	222 (71.6)	1 (1.1)	0.03–6.3	+0% (−5.1–5.1, *p* = 1)
After fourth vaccine			1 (1.1)	0.03–6.2	
Before fifth vaccine	240	17 (6.6)	1 (0.4)	0.01–2.3	−0.4% (−2.3–1.2, *p* = 1)
After fifth vaccine			0 (0.0)	0.0–1.5	

*pp* percentage points.

^a^Missing samples at each dose represents participants who received the respective vaccine but were excluded from analyses if vaccination occurred less than 180 days after a previous dose.

## Discussion

In this nationwide study, we included a large cohort of LTx recipients, on maintenance immunosuppression, that were offered vaccination against COVID-19 with BNT162b2 vaccine. We determined prevalence and relative incidence of ALT elevation and use of biopsies. We found no significant differences in prevalence of elevated ALT before and after BNT162b2 vaccination, and we found no evidence of increased risk of elevated ALT after vaccination. Furthermore, we observed no differences in prevalence of elevated ALT above CTCAE grade 1. Lastly, we found no difference in the prevalence of liver biopsies before and after any BNT162b2 doses.

We followed 393 LTx recipients for more than three years and included information on up to seven vaccine doses in a real-world setting and observed no change in the relative incidence or prevalence of elevated ALT after BNT162b2 vaccination. These results corroborate previous findings in SOT recipients after two doses of either BNT162b2 or ChAdOx1^20^, and further indicate that repeated vaccination is not associated with episodes of elevated ALT.

ALT is released upon hepatocellular damage, making it a sensitive biomarker for liver injury. The etiologies of ALT elevations are diverse and causes for acute ALT elevation are conditions such as hepatic ischemia, drug-induced injury, or acute viral hepatitis^21^. We observed 70 episodes of ALT elevations of grade ≥2, which could indicate of graft injury. Usually, ALT elevations of grade ≥2 in LTx recipients will lead to increased surveillance and further diagnostics depending on the overall clinical picture. As we do not have data on the specific causes of ALT elevation in this study, it is important to keep in mind that in LTx recipients, elevated ALT alone is not sufficient to diagnose acute graft rejection, and elevated ALT must be interpreted as part of the entire clinical picture.

Similar to elevated ALT measurement, we observed no difference in the prevalence of biopsies performed on clinical indication before and after vaccination with BNT162b2. A liver biopsy is the golden standard for diagnosing acute graft rejection^19^, and while percutaneous ultrasound guided liver biopsies are associated with low incidence of serious adverse events^22^, the procedure is not without risk, with bleeding being the most important complication^23^. Thus, our finding is reassuring and raises no concerns about an association between vaccination with BNT162b2 and rejection episodes or unnecessary clinical interventions in LTx recipients.

To investigate the potential for confounding due to SARS-CoV-2 infection status, we investigated the incidence rate of positive SARS-CoV-2 PCR tests before and after vaccination with BNT162b2. We found a higher incidence rate in the periods after vaccination than in the periods before, primarily due to a high number of cases in the period after the fourth vaccine dose. This period coincided with the peak of incidence of infections with the SARS-CoV-2 Omicron variant in Denmark during January and February 2022. Since SARS-CoV-2 infection is associated with ALT elevations and infections were more common post-vaccination, confounding would likely enhance rather than mask a signal. Although some asymptomatic infections may remain uncaptured, we find no reason for concern with regards to underestimating a vaccination related effect on ALT elevations due to confounding from SARS-CoV-2 infection status.

Contrary to our hypothesis, our findings do not support an association between elevated ALT and no increased use of liver biopsies after BNT162b2 vaccination in LTx recipients. Our study provides important evidence on the safety of BNT162b2 vaccination for health authorities and transplant clinicians to guide future vaccination recommendations. Considering the well-established benefits of vaccination in preventing severe COVID-19 and death^3–5^, this study provides support to the current vaccination strategy to prevent severe COVID-19 in LTx recipients.

It is a strength that our study was conducted on LTx recipients included in the nationwide, prospective, and well-characterized the Danish Comorbidity in Liver Transplant Recipients (DACOLT) study. Another strength is the use of the Danish vaccination register (DDV), the Danish microbiology database (MiBa), and the Danish pathology data bank (DPDB), which are national databases that provide robust and complete data on vaccination, SARS-CoV-2 infections, and biopsies for the cohort. Furthermore, follow-up was more than three years and included information on up to seven vaccine doses in a real-world setting.

Limitations include comparing periods before the first vaccine to after the second dose for the prevalence estimates. The requirement for both an ALT measurement 90 days before and after a vaccination and excluding subsequent vaccines with less than 180 days apart resulted in missing data in the estimates of prevalence of ALT elevation. Importantly, this was not the case for the SCCS analysis, where data was included even when risk periods overlapped. In these analyses, overlapping exposure periods are handled by splitting the observation time into separate intervals with clear exposure status, ensuring no data loss. Using routine clinical care data may have led to unbalanced sampling bias, as participants with higher disease burden may be sampled more frequently. Additionally, we assumed that participants without a baseline ALT measurement had a normal ALT at baseline. A large proportion of participants lacked a baseline ALT measurement, and we cannot rule out that we some of the participants without baseline ALT measurements may have had abnormal ALT. As the definition of elevated ALT rely on whether baseline ALT is normal or abnormal this could lead to misclassification of events as being elevated ALT, although they are not. However, we would expect this misclassification to be evenly distributed between post vaccination risk periods and control periods, thus lowering the risk of bias. Furthermore, we did not investigate other possible reasons for elevated ALT e.g., diseases or surgical procedures or investigate other liver function tests than ALT. Finally, most SOT recipients in Denmark were vaccinated with BNT162b2, and we censored participants when they received other COVID-19 vaccines than BNT162b2. Thus, our results are not generalizable to populations with more heterogenous COVID-19 vaccination schedules.

In conclusion, we found no evidence to support an association between elevated ALT or increased use of liver biopsies after BNT162b2 vaccination in LTx recipients.

## Methods

### Study design

This cohort study is a sub-study of the DACOLT study^24^. The DACOLT study is an ongoing nationwide, prospective cohort study that aims to investigate the burden of comorbidities in LTx recipients. All living recipients above 20 years of age followed at an outpatient clinic in Denmark and able to provide informed content are invited to participate in the study.

In Denmark, the COVID-19 vaccination program was initiated on December 27^th^, 2020^25^. In this study, follow-up started three months before the administration of the first vaccine dose for each participant. If a participant did not receive any vaccines, follow-up started December 27^th^, 2020. Participants had to be at least one year from transplantation at the start of follow-up to be eligible for inclusion, as we deemed to include participants who were in stable immunosuppressive maintenance therapy.

The end of follow-up was May 31^st^, 2024, death, re-transplantation, or administration of a COVID-19 vaccine other than monovalent or bivalent BNT162b2 vaccines, whichever came first. This study did not influence the vaccination strategy of the participants. All blood samples and liver biopsies were performed as part of clinical routine or based on clinical indication as a part of routine clinical care. LTx recipients in Denmark are monitored with ALT measurements at least every six months.

The DACOLT study (clinical trial identifier NCT04777032) is approved by the Committee on Health Research Ethics of The Capital Region of Denmark (approval number H-20052199).

### Data collection

Clinical information, including demographics, ALT measurement dates and values, date and reason for transplantation, date of re-transplantation, use of immunosuppressive medication at inclusion in DACOLT study, acute graft rejections, and time of death were collected from medical records.

Information regarding COVID-19 vaccine types and administration dates were acquired from DDV. It has been mandatory to register all vaccines administered in Denmark in DDV since 2015^26^. Data on liver biopsies were collected from DPDB, which contains all information on biopsies performed in Denmark since 1990^27^. Information on SARS-CoV-2 infections was retrieved from MiBa, a nationwide database that encompasses all SARS-CoV-2 PCR tests from the primary healthcare sector, hospitals and test centers across Denmark^28^. Liver biopsies included in this study were categorized using information from medical records as either pre-scheduled protocol biopsies or biopsies performed on clinical indication.

### Definitions

We defined baseline ALT for each participant as the median of ALT measurements from September 27^th^, 2020, to three months before administration of the first vaccine dose in each participant. For participants who did not receive any vaccines, baseline ALT was defined as the median of ALT measurements from September 27^th^, 2020, to December 27^th^, 2020, when the first COVID-19 vaccine was administered in Denmark. Participant who did not have a baseline ALT measurement (*n* = 149, 37.9%) were considered to have a normal ALT at baseline. Baseline ALT was categorized as elevated in accordance with the Danish Health Authorities’ definition of upper limits of normal (ULN)^29^.

Severity of elevated ALT was categorized according to CTCAE 5.0^30^. According to CTCAE, an elevated ALT measurement is categorized as grade 1 ( >ULN – 3.0 x ULN if baseline was normal; 1.5–3.0 x baseline if baseline was abnormal), grade 2 ( >3.0–5.0 x ULN if baseline was normal; >3.0 – 5.0 x baseline if baseline was abnormal), grade 3 ( >5.0–20.0 x ULN if baseline was normal; >5.0–20.0 x baseline if baseline was abnormal) and grade 4 ( >20.0 x ULN if baseline was normal; >20.0 x baseline if baseline was abnormal).

We defined biopsies on clinical indication as biopsies performed due to suspicion of rejection or suspicion of de novo liver-disease by a transplant clinician.

In our liver transplant centre, first choice treatment of acute rejections is high-dose methylprednisolone. Thus, we defined acute graft rejection as a biopsy-confirmed acute rejection, treated with high-dose methylprednisolone for 3–5 days.

### Self-controlled case series analysis

To investigate the relative incidence of ALT elevation after COVID-19 vaccinations, we used a SCCS analysis. All participants who had at least one elevated ALT measurement during the follow-up were included in the SCCS analysis. If a participant had multiple elevated ALT measurements, they were included as separate cases in the SCCS analysis if at least one normal ALT sample separated each elevated ALT. In the SCCS analysis, we defined risk periods as 21 days after the first vaccination and 90 days for the remaining vaccine doses. The 90-day interval was a pragmatic choice made in order to reduce the risk of missing episodes of increased ALT or biopsies. We chose 90 days as the recommended minimum time between COVID-19 vaccine booster doses in Denmark was three months, and as we have previously found the immune response to peak in SOT recipients after three months^3^. The risk period of 21 days after the first vaccine dose was chosen based on a clinical rationale, as the recommended interval between the first and second vaccine doses was 21 days. Control periods were defined as time not defined as risk periods. In the SCCS analysis, follow-up started three months before the administration of the first vaccine dose for each participant. If a participant did not receive any vaccines, follow-up started December 27^th^, 2020.

### Statistics

Statistical analyses were performed using R (4.3.2). Continuous data were reported as medians with IQR. Categorical data were reported numerically and as percentages.

**Fig. 3:**
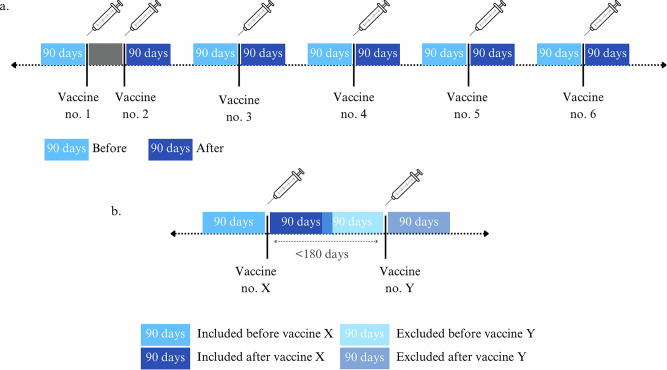
Prevalence of elevated ALT or liver biopsies in the 90 days before a vaccine is compared to 90 days after a vaccine. **a** The period 90 days before the first vaccine is compared to 90 days after the second vaccine. Prevalence of elevated ALT or liver biopsies are not calculated in the period between the first and second vaccine. The grey area between vaccine doses one and two indicates a 21-day interval. **b** If there were less than 180 days between two vaccination dates, ALT samples or liver biopsies did not contribute to the before period of the following vaccine and, thus prevalence of elevated ALT or liver biopsies could not be calculated for the following vaccine.

Prevalence of elevated ALT and liver biopsies were calculated as the number of participants with an elevated ALT measurement or a liver biopsy 90 days before and 90 days after each COVID-19 vaccine dose, except for the prevalence 90 days before the first vaccine which was compared to the prevalence 90 days after the second vaccine (Fig. 3A).

**Fig. 4:**
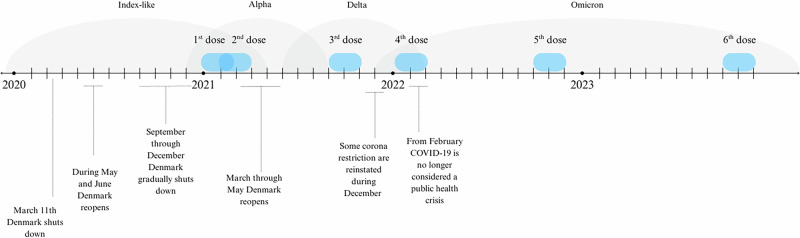
The timeline illustrates the interplay of vaccine administration periods, the dominant SARS-CoV-2 variants in Denmark and the dynamic progression of COVID-19 lockdowns and subsequent lifting of restrictions. Blue boxes on the timeline indicate when most LTx recipients in the study received their respective vaccine. Above the timeline, the prevailing SARS-CoV-2 variant in Denmark is indicated at each given time point. Below the timeline, major COVID-19 lockdowns and the reopening of society are indicated.

Participants were included in the analyses of prevalence of elevated ALT, if they had at least one ALT sample both 90 days before and 90 days after administration of a COVID-19 vaccine, resulting in a complete-case analysis. If less than 180 days separated two vaccination dates and the 90 days after vaccination overlapped with the 90 days before the following vaccine, the prevalence was not calculated for the before- and after-period for the latter of the two vaccines (Fig. 3B). An overview of the interplay between vaccine administration periods, the dominant SARS-CoV-2 variants in Denmark, and the dynamic progression of COVID-19 lockdowns and subsequent lifting of restrictions can be found in Fig. 4.

Prevalence of elevated ALT and liver biopsies were calculated with 95% CI using the exact method. Difference in prevalence was calculated in percentage point (pp) with 95% CI and was tested using McNemar’s test.

The incidence rate of positive SARS-CoV-2 PCR test 90 days before and after vaccination with BNT162b2 was calculated by dividing the number of cases with person-time at risk, and 95% CI were calculated using Byar’s approximation.

In a sensitivity analysis, all analyses were repeated after excluding participants who were censored due to receiving other COVID-19 vaccines than BNT162b2.

## Supplementary information

Supplementary Information

## Data Availability

Due to the sensitive nature of the research and the potential for participant re-identification, the datasets generated and analyzed during the current study are not publicly available. This includes all individual de-identified participant data, as even with de-identification, the risk of compromising participant confidentiality remains. Consequently, no data or additional related documents will be shared.

## References

[1] Anjan, S. et al. Is the Omicron variant truly less virulent in solid organ transplant recipients?. *Transpl. Infect. Dis.* **24**, e13923 (2022). 35915957 10.1111/tid.13923 PMC9538470

[2] Ma, E. et al. Omicron infections profile and vaccination status among 1881 liver transplant recipients: a multi-centre retrospective cohort. *Emerg. Microbes Infect.* **11**, 2636–2644 (2022). 36227753 10.1080/22221751.2022.2136535 PMC9639509

[3] Hamm, S. R. et al. Decline in antibody concentration 6 months after two doses of SARS-CoV-2 BNT162b2 vaccine in solid organ transplant recipients and healthy controls. *Front Immunol.* **13**, 832501 (2022). 35281023 10.3389/fimmu.2022.832501 PMC8905653

[4] Naylor, K. L. et al. Effectiveness of a fourth COVID-19 mRNA vaccine dose against the omicron variant in solid organ transplant recipients. *Transplantation* **108**, 294–302 (2024). 38098159 10.1097/TP.0000000000004766

[5] Callaghan, C. J. et al. Vaccine effectiveness against the SARS-CoV-2 B.1.1.529 omicron variant in solid organ and islet transplant recipients in England: a national retrospective cohort study. *Transplantation* **107**, 1124–1135 (2023). 36727724 10.1097/TP.0000000000004535 PMC10125014

[6] Villavicencio, A. et al. Adverse events after SARS-CoV-2 vaccination in solid organ transplant recipients: a systematic review. *Transpl. Infect. Dis.* **24**, e13936 (2022). 36067062 10.1111/tid.13936 PMC9538206

[7] Davidov, Y. et al. Immunogenicity and adverse effects of the 2-dose BNT162b2 messenger RNA vaccine among liver transplantation recipients. *Liver Transplant.* **28**, 215–223 (2022). 10.1002/lt.26366 PMC8661838 34767690

[8] Luo, X., Lessomo, F. Y. N., Yu, Z. & Xie, Y. Factors influencing immunogenicity and safety of SARS-CoV-2 vaccine in liver transplantation recipients: a systematic review and meta-analysis. *Front Immunol.* **14**, 1145081 (2023). 37731498 10.3389/fimmu.2023.1145081 PMC10508849

[9] Centers for Disease Control and Prevention. Vaccines for Moderately to Severely Immunocompromised People. https://www.cdc.gov/covid/vaccines/immunocompromised-people.html (accessed July 15, 2025).

[10] Sundhedsstyrelsen. Vaccination mod influenza og covid-19. https://www.sst.dk/da/vaccination (accessed July 15, 2025).

[11] World Health Organization. COVID-19 advice for the public: Getting vaccinated. https://www.who.int/emergencies/diseases/novel-coronavirus-2019/covid-19-vaccines/advice (accessed July 15, 2025).

[12] Vyhmeister, R., Enestvedt, C. K., VanSandt, M. & Schlansky, B. Steroid-resistant acute cellular rejection of the liver after severe acute respiratory syndrome coronavirus 2 mRNA vaccination. *Liver Transplant.* **27**, 1339–1342 (2021). 10.1002/lt.26097 PMC8242872 33993619

[13] Sarwar, R., Adeyi, O. A., Lake, J. & Lim, N. Acute cellular rejection in liver transplantation recipients following vaccination against coronavirus disease 2019: a case series. *Liver Transplant.* **28**, 1388–1392 (2022). 10.1002/lt.26446 PMC9088651 35243757

[14] Okamoto, T. et al. Two cases of possible exacerbation of chronic rejection after anti-SARS-CoV-2 messenger RNA vaccination: a case report. *Transpl. Proc.* **55**, 530–532 (2023). 10.1016/j.transproceed.2022.11.009 PMC9708617 36572611

[15] Hume, S. J., Jackett, L. A., Testro, A. G., Gow, P. J. & Sinclair, M. J. A case series of patients with acute liver allograft rejection after anti-SARS-CoV-2 mRNA vaccination. *Transplantation* **106**, E348–E349 (2022). 35427294 10.1097/TP.0000000000004166 PMC9213051

[16] Hughes, D. L. et al. Guillain-Barré syndrome after COVID-19 mRNA vaccination in a liver transplantation recipient with favorable treatment response. *Liver Transplant.* **28**, 134–137 (2022). 10.1002/lt.26279 PMC8661837 34431208

[17] Mahalingham, A., Duckworth, A. & Griffiths, W. J. H. First report of post-transplant autoimmune hepatitis recurrence following SARS-CoV-2 mRNA vaccination. *Transpl. Immunol.* **72**, 101600 (2022). 35390478 10.1016/j.trim.2022.101600 PMC8977213

[18] Dumortier, J. Liver injury after mRNA-based SARS-CoV-2 vaccination in a liver transplant recipient. *Clin. Res. Hepatol. Gastroenterol.* **46**, 101743 (2022). 34146727 10.1016/j.clinre.2021.101743 PMC8214934

[19] Krenzien, F. et al. Diagnostic biomarkers to diagnose acute allograft rejection after liver transplantation: systematic review and meta-analysis of diagnostic accuracy studies. *Front Immunol.* **10**, 758 (2019). 31031758 10.3389/fimmu.2019.00758 PMC6470197

[20] Ajlan, A. A. et al. Comparison of the safety and immunogenicity of the BNT-162b2 vaccine and the ChAdOx1 vaccine for solid organ transplant recipients: a prospective study. *BMC Infect. Dis.* **22**, 786 (2022). 36229772 10.1186/s12879-022-07764-x PMC9559153

[21] Melendez-Rosado, J., Alsaad, A., Stancampiano, F. F. & Palmer, W. C. Abnormal liver enzymes. *Gastroenterol. Nurs.* **41**, 497–507 (2018). 30418344 10.1097/SGA.0000000000000346

[22] Mulazzani, L. et al. Retrospective analysis of safety of ultrasound-guided percutaneous liver biopsy in the 21st century. *Eur. J. Gastroenterol. Hepatol.* **33**, E355–E362 (2021). 35048647 10.1097/MEG.0000000000002080

[23] Rockey, D. C., Caldwell, S. H., Goodman, Z. D., Nelson, R. C. & Smith, A. D. Liver biopsy. *Hepatology* **49**, 1017–1044 (2009). 19243014 10.1002/hep.22742

[24] Thomsen, M. T. et al. The Danish comorbidity in liver transplant recipients study (DACOLT): a non-interventional prospective observational cohort study. *BMC Gastroenterol.* **21**, 145 (2021). 33794793 10.1186/s12876-021-01733-5 PMC8017840

[25] Statens Serum Institut. Tidslinje for covid-19. https://www.ssi.dk/-/media/arkiv/subsites/covid19/presse/tidslinje-over-covid-19/covid-19-tidslinje-for-2020-2022-lang-version---version-1---april-2022.pdf (accessed July 15, 2025).

[26] Krause, T. G., Jakobsen, S., Haarh, M. & Mølbak, K. The Danish vaccination register. *Eurosurveillance* **17**, 20155 (2012). 22551494 10.2807/ese.17.17.20155-en

[27] Bjerregaard, B. & Larsen, O. B. The Danish pathology register. *Scand. J. Public Health* **39**, 72–74 (2011). 21775357 10.1177/1403494810393563

[28] Voldstedlund, M., Haarh, M. & Mølbak, K. The Danish Microbiology Database (MiBa) 2010 to 2013. *Eurosurveillance* **19**, 20667 (2014). 24434175 10.2807/1560-7917.es2014.19.1.20667

[29] Sundhedsstyrelsen. Lægehåndbogen: ALAT. https://www.sundhed.dk/sundhedsfaglig/laegehaandbogen/undersoegelser-og-proever/klinisk-biokemi/blodproever/alat/ (accessed July 15, 2025).

[30] National Cancer Institute (USA). Common Terminology Criteria for Adverse Events (CTCAE) Version 5.0. https://dctd.cancer.gov/research/ctep-trials/for-sites/adverse-events/ctcae-v5-5x7.pdf (accessed July 15, 2025).

